# Change of Use in Community Services among Disabled Older Adults during COVID-19 in Japan

**DOI:** 10.3390/ijerph18031148

**Published:** 2021-01-28

**Authors:** Tomoko Ito, Sachiko Hirata-Mogi, Taeko Watanabe, Takehiro Sugiyama, Xueying Jin, Shu Kobayashi, Nanako Tamiya

**Affiliations:** 1 Department of Health Services Research, Faculty of Medicine, University of Tsukuba, Tsukuba, Ibaraki 305-8577, Japan; tito@md.tsukuba.ac.jp (T.I.); kinnsetuei@yahoo.co.jp (X.J.); ntamiya@md.tsukuba.ac.jp (N.T.); 2 Health Services Research and Development Center, University of Tsukuba, Tsukuba, Ibaraki 305-8577, Japan; sachiko-hirata@bm-sms.co.jp (S.H.-M.); taeko-watanabe@umin.ac.jp (T.W.); 3 Analytics & Innovation Department, Research & Development Group, SMS Co., Ltd., Tokyo 105-0011, Japan; shu-kobayashi@bm-sms.co.jp; 4 Diabetes and Metabolism Information Center, Research Institute, National Center for Global Health and Medicine, Tokyo 162-8655, Japan

**Keywords:** COVID-19, older adults, community services, home-visit services, outpatient services

## Abstract

During the COVID-19 pandemic, social interactions were restricted, including community services for disabled older adults. This study aimed to describe the change of use in community services related to long-term care insurance (LTCI) during the pandemic in Japan. A retrospective descriptive study was conducted using data collected via a cloud-based management support platform for older adult care provider “*Kaipoke*”, by a private-sector company “SMS Co., Ltd.”, in which care-managers of LTCI manage their office work. Data collection occurred from July 2019 to June 2020. Study subjects were LTCI service users aged 65 years and above. Subjects were living at home. We examined changes in the number of users of LTCI services before and after the COVID-19 pandemic began, using an interrupted time-series analysis. Results indicated that the use of outpatient services was reduced; however, home-visit services were maintained. The decrease in use was significant in the seven prefectures where the infection initially spread. There are concerns that older adults or surrounding caregivers can be affected by such changes in LTC service use. It is therefore necessary to implement sustainable measures from a long-term perspective and investigate their influence as part of future studies.

## 1. Introduction

In efforts to prevent the proliferation of the novel coronavirus disease 2019 (COVID-19), citizens were instructed to “stay at home” or “isolate in place” by government officials worldwide. People were forced to refrain from going out, to stop attending school, and to work from home.

In Japan, the first infection was recorded on 16 January 2020. The number of infected people gradually increased between January and March 2020 [1], especially among returnees from foreign countries. On 7 April, the government subsequently issued a declaration of emergency across seven prefectures where the infection had spread [2]. The declaration was accompanied by a recommendation to reduce contact with others by 70%; people were urged to stay at home. The state of emergency was then expanded to all prefectures, which called for further infection prevention measures.

Under these circumstances, older adults also broadly prevented themselves from going out and restricted their activities. Reports indicate that older adults are particularly vulnerable to COVID-19 and must avoid the risk of infection [3,4]. Moreover, it is also reported that frail older adults with chronic diseases are particularly susceptible to severe conditions of the disease. Clinically, older adults requiring long-term care (LTC) are more likely to have complicated comorbidities and are a high-risk population for COVID-19 [5]. Globally, there have been reports of the pandemic spreading in nursing homes for frail, older adults. Nursing homes have been closed and visitors of the residents have been turned away. The staff live in fear, taking every precaution for their residents. The world has been quick to pay attention to infection control in nursing homes. Many cases have been reported and discussed. However, there has been little discussion on the status and condition of frail older adults living in the community.

In Japan, long-term care insurance (LTCI) services are provided both for residents in nursing homes and for community-dwelling older adults with disability. For LTCI services, 6.45 million older adults (≥65 years old) were certified as users in March 2019 [6]. Of these, there are overwhelmingly more community-dwelling users than residents in nursing homes, by about four times as many. Community-dwelling users can avail several types of services, such as home-visit services and outpatient services [7] as a necessary part of daily life. Previous studies have reported on the effectiveness of LTCI service use for community-dwelling older adults. However, the COVID-19 pandemic has forced them to limit their usage of LTCI services.

As a matter of concern, we need to consider the occurrence of various adverse events due to reduced service use. In general, restriction of activity and social isolation are risk factors for the deterioration of physical and psychiatric functions and mortality [8,9,10,11]. During the COVID-19 pandemic, these were the “second” most serious health risk factors among older adults, with international reports cautioning against such health hazards in this age group [12,13,14,15].As a primary health outcome, symptoms, serious conditions, and death due to COVID-19 are urgent adverse events that must be monitored in a timely manner. On the other hand, lifestyle changes caused by the pandemic and their longitudinal outcomes are also an important public health concern. These adverse events are a long-term consequence and can be observed only later. Therefore, now it is important to empirically describe the highly unusual changes brought about by COVID-19 as a foundation for future studies. We need to capture the resulting changes and to prepare for future research regarding the impact. Previous experimental research has not indicated actual restrictions in care for community-dwelling older adults with disability, including in Japan. Therefore, this study aimed to describe the change of use in community services relating to LTCI amid the COVID-19 pandemic in Japan. The research hypothesis is that, owing to the pandemic, the use of services has reduced, especially in outpatient services and services for mildly disabled older adults; we assumed that services with higher risk of infection and those with less urgent necessity for care have reduced significantly.

## 2. Materials and Methods

### 2.1. Data Source

The data in the present study were collected via a cloud-based management support platform for older adults care providers called “*Kaipoke*”, offered by the private-sector company “SMS Co., Ltd.” (“SMS”). The *Kaipoke* system helps LTCI service providers, including care-managers, to manage their office functions. Individuals who are certified as having a disability and requiring in-home care are assessed regarding their care needs by LTCI care managers, who arrange the use of their services. The collected data included the type of service rendered, demographic data, and the care requirements of the patients. The *Kaipoke* system has been implemented in numerous care-manager offices in each prefecture, making it possible to assess trends in LTCI service use almost nationwide. Data collection occurred over 12 months, from July 2019 to June 2020; we included data before January 2020, when the first domestic COVID-19 infection was confirmed, to compare the trend before and after the pandemic.

### 2.2. Study Subjects

The study subjects were collected based on LTCI claims data. Generally, users of LTCI services are certified for care requirements divided into two major categories: support level (SL) and care level (CL). The SL is the level where the subject mainly needs to be looked after, while CL is the level where the subject needs to be cared for; CL requires a higher level of care. Those with SL mainly need physical training or watching to prevent future deterioration. Therefore, available services are clearly different when comparing SL and CL. In addition, the certification criteria differ for subjects under the age of 65. After being certified by the LTCI system, a care plan is created with a care manager in order to use the service. In some cases, the service is not used according to the plan. There may be some difference between the plan and usage data.

In accordance with the above situation, the present study’s subjects were selected with inclusion/exclusion criteria as follows. First, the data were limited to subjects living in a community, not in nursing homes, because the *Kaipoke* system is offered only for them. Second, subjects certified as SLs were excluded because of the difference in available services. Next, subjects under 65 years were excluded. Finally, only data on service usage were analyzed to capture the actual situation.

### 2.3. Ethics Approval and Consent to Participate

This study was approved by the Medical Ethics Review Board of the University of Tsukuba (approval number 1301-4). As this study was retrospective in design and had no data identifying individuals, informed consent and opt-out were not possible. These are methods based on the ethical guidelines for research [16].

### 2.4. Measures

The primary measure was the number of people who used LTCI services each month. Demographic variables comprised the age and sex of subjects. The care requirement level was measured by the care needs of the certified persons defined by the LTCI system in Japan. The levels ranged from care level (CL)1 to CL5, with CL5 being for patients who were severely ill and almost bedridden. In CL1, patients were able to walk using a cane or other assistance, but required some daily care. People with milder disabilities than CL1 who qualified for the use of LTCI prevention services were excluded from this study, since they were managed in a different way than people with CLs 1 to 5. The care level is determined by the estimated amount of care time needed [17]; roughly, those with CLs 4 or 5 are almost bedridden, while CLs 1 to 3 are not. Reports based on the national survey of LTCI service providers about the kind of care provided reveal [18] that those with CLs 4 or 5 mainly received services for toileting, eating, and bathing while those with CLs 1 to 3 often received services for house cleaning, laundry, or watchful waiting assistance. As a geographic variable, the prefecture where the user resided was included because the Japanese government issued its declaration of emergency on a prefecture-by-prefecture basis. The prefecture is Japan’s first level of administrative division; Japan has 47 prefectures which have their own local governments and assemblies. In this study, all 47 prefectures in Japan were divided into two categories: the seven prefectures where a state of emergency was declared in April 2020, and the others (35 prefectures).

Finally, the service use measure included the use of all types of LTCI services and was classified by type of service. Service types included home-visit care, home-visit nursing, home-visit bathing, outpatient care, and outpatient rehabilitation. Home-visit care supported daily life and included assistance with toileting and preparation of meals. Home-visit nursing consisted of administering medical treatment by a nurse. Home-visit bathing involved bringing a bathtub and preparing for bathing, which required special equipment and multiple personnel. Outpatient care and outpatient rehabilitation are services that are conducted at a facility. Outpatient care included a program to help people with daily tasks, such as bathing and eating, and the enjoyment of recreational activities with other users. Outpatient rehabilitation comprised training by a therapist for functional recovery and maintenance.

### 2.5. Statistical Analysis

The outcome was the trend in the number of monthly users for LTCI services (Y_t_). Trend was assessed using an interrupted time-series analysis. In the model, the periods were divided into pre-COVID-19 (July 2019–December 2019) and post-COVID-19 (January 2020–June 2020). The results were expressed by three coefficients: β_T_, β_Xt_, and β_TXt_, where T is the time elapsed each month (1 ≤ T ≤ 12) from the start of observation (July 2019) to the end of observation (June 2020). Xt is a dummy variable as a dichotomous variable, including pre-COVID-19 (Xt = 0) and post-COVID-19 (Xt = 1) [19].

Y_t_ = β_0_ + β_T_T + β_Xt_Xt + β_TXt_TXt + e_t_

β_0_: estimates the base level

β_T_: estimates the trend pre-COVID-19.

β_Xt_: estimates the change in level post-COVID-19

β_TXt_: estimates the change in trend post-COVID-19

According to this formula, the generalized linear model was applied with a log link and Poisson distribution. The models were also tested in groups stratified by sex, age, care requirement level, and region. STATA version 14.2 (Stata-Corp LP, College Station, TX, USA) was used for the analysis. The figures were output via the STATA syntax “itsa” introduced by Linden [20]. Additionally, the estimated trend (persons per month) was calculated by this syntax.

Furthermore, because a different trend after the pandemic was observed, extra analysis excluding the data in June 2020 was conducted as a sensitivity analysis. The statistical significance level was a two-sided *p*-value of less than 5%.

## 3. Results

Table 1 shows the number of LTCI service users observed during the six months of July–December 2019 for Pre- and January–June 2020 for Post-COVID-19. In the case of all service users, the number of users gradually increased from the previous month in all months except for May 2020. In May 2020, the number of users decreased by 553 compared to the previous month, April 2020. However, the increase was different between Pre- and Post- COVID-19, averaging 7429 users in Pre- and 6389 users in Post-COVID-19.

The characteristics of each user were then described in terms of sex, age, care need level, and whether they lived in the seven prefectures or not. In terms of sex, there was no obvious change in the percentage of male users, ranging from 36.4% to 36.6%. The mean age was 83.9 years old (SD 7.7), but the users were getting older, as the percentage of users aged 85 years and above increased from 49.2% in Pre- to 52.5% in Post-COVID-19. By care need level, there was no major change in the trend of the percentage of users. Finally, the percentage of users residing in the seven prefectures increased slightly in each month in Pre-COVID-19, but began to decrease slightly in Post-COVID-19 from March to May 2020.

In Table 2, the trend of the number of users was analyzed by ITSA for the two periods, Pre- and Post-COVID-19. The trend Pre-COVID-19 in the overall number of users showed an increase (β = 0.0120, *p* < 0.001). Post-COVID-19, the trend changed to negative (*p* < 0.001). This negative trend was more pronounced for two outpatient services users: outpatient care (β = −0.0129, *p* < 0.001) and outpatient rehabilitation (β = −0.0128, *p* < 0.001). However, for home-visit bathing or home-visit nursing users, and CL4 or CL5 users, an increasing trend was seen in spite of the spread of COVID-19. In particular, home-visit bathing showed a larger increase (β = 0.0188, *p* < 0.001) than before the COVID-19 spread.

The result with coefficient (β) of the regression model was translated into an estimated number of users as shown in Table A1. We obtained significant estimates Post-COVID-19 only for two types of outpatient services (outpatient care and outpatient rehabilitation) and home-visit bathing. Outpatient care and rehabilitation users saw a decrease Post-COVID-19 reversing the pace of increase during Pre-COVID-19: outpatient care −2468 (95% confidential interval −4542.0 to −395.4) and outpatient rehabilitation −533.1 (−1016.7 to −49.6). For home-visit bathing, the pace of increase in Pre-COVID-19 was 94.8 (30.6 to 159.1); however, this was amplified to 208.4 in Post-COVID-19 (120.0 to 296.8).

**Figure 1:**
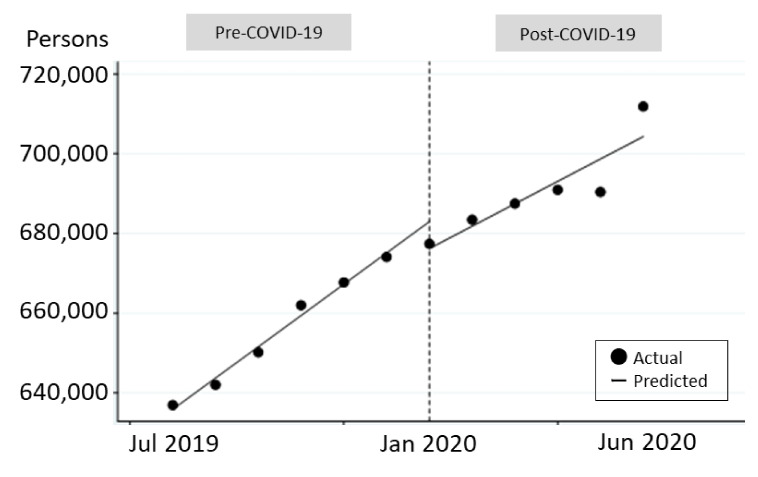
Trends of users for all services.

These changes in trend between the two periods of Pre-COVID-19 and Post-COVID-19 in Table 2 are also shown visually in the figures with the observed number of users in dots and the estimated approximation in lines. In Figure 1, the trend of a gradual increase was maintained Pre-COVID-19; however, it was disturbed Post-COVID-19. The observed number of users was scattered without a smooth tendency, especially dropping in May 2020 and recovering in June.

**Figure 2:**
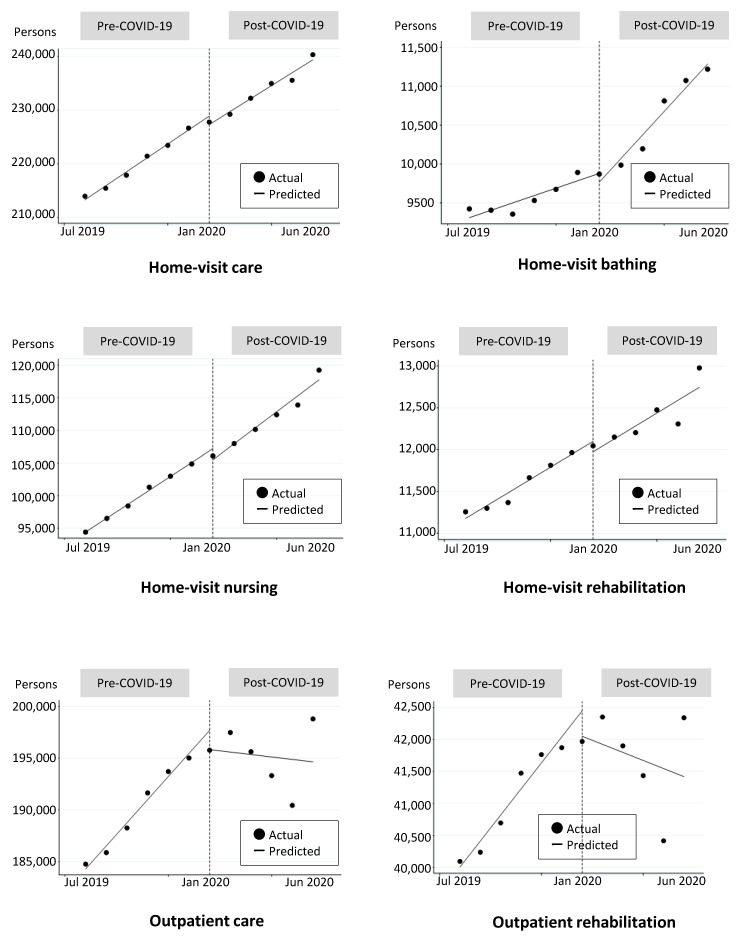
Trends of users for each service.

These disturbing trends are also observed in Figure 2 by type of service, especially for outpatient care and outpatient rehabilitation. The estimated line Post-COVID-19 shows a clear rightward trend in the two types of outpatient services. These are seen as convex trend changes. On the other hand, the figure for home-visit bathing shows a concave trend change, hollowed out at the bottom.

The results of sensitivity analysis excluding June were similar to the main results (shown in Table A2). However, for home-visit nursing, a downward trend was observed in contrast to the main results.

## 4. Discussion

This is the first study to describe the changes in the use of community services for LTCI in Japan under the spread of COVID-19. Analysis of nationwide data showed an overall change in services used during the COVID-19 pandemic. Specifically, the decrease in service use was concentrated in outpatient services among the mildly disabled older adults, in areas with a high spread of infection.

Outpatient service users were much decreased after the pandemic: the 2468 users for outpatient care and 533 users for outpatient rehabilitation were estimated to decrease monthly, comparable to the rate of increase before the pandemic. It was expected that the use of outpatient services would be suppressed due to the preventive measure of limiting gatherings. This was implemented nationwide to curb the spread of infection [21] and was strictly observed in the LTC services because older adults are a high-risk population for COVID-19 [4]. Thus, the use of services was restricted in the seven prefectures where the declaration of emergency was first issued. On the other hand, the restriction of home-visit services was less than that of outpatient care. This is attributed to the nature of home-visit services, which provide assistance with essential daily tasks. However, the use of home-visit rehabilitation was more limited than other home-visit services. This may be because home-visit rehabilitation was less essential to daily living. Similarly, the use of services by older adults with mild disabilities in CLs 1 to 3 may not be as urgent as those in CLs 4–5. Conceivably, their lives were somehow helped by their family. It was also expected that home-visit nursing would be a crucial service. Finally, only the use of home-visit bathing increased. Before the pandemic, the estimated number of users had been growing monthly, but this rate increased dramatically in the months following the outbreak of the pandemic. These increases were likely due to the reduction of outpatient services use, since most users of outpatient services required assistance with bathing at the facility [22]. Results suggested that COVID-19 changed the way LTCI services were used, replacing some outpatient services with home-visit services instead. Additionally, as a result of the sensitivity analysis, even the data excluding June 2020 showed a significant decreasing trend. However, the increase in June 2020 may be a reaction to the previously restrained use of services which may have entered a different phase, in that the state of emergency was lifted on 25 May for all of Japan. Such relaxation of regulations may have led to a recovery in the use of services, which should be investigated in a future study.

As for the change of trend between Pre- and Post-COVID-19, older adults continuingly used essential services while home-visit services substituted for some outpatient services. Among other examples, the increase in home-visit bathing could show this. In the Japan LTCI system, nurses should take part in home-visit bathing and deliver treatments for users [23]. They observe users’ physical conditions or bedsores when needed. The increase in home-visit nursing might have the same significance. However, some of the lost outpatient service functions could not be compensated for by the substitution of other services. As stated in the introduction, older adults could lose opportunities to maintain group activities and interactions.

These changes in LTCI service use, especially in outpatient services, may lead to a variety of future adverse events. One of these is frailness, which weakens the physical and psychiatric functioning of older adults and reduces their ability to engage in daily activities from a longitudinal perspective. A previous study also argues that daily activities can be related to psychological well-being [24]. Amid the COVID-19 pandemic, many researchers have noted these risks for the future [13,14,15,25,26]. Presently, the control measures implemented for COVID-19 are expected to continue. This pandemic has caused lifestyle changes and restrictions in activities and gatherings, which are likely to be imposed for a long time. Furthermore, the importance of in-home physical activity for older adults has been mentioned in global communications [27]. It was also noted that, in the absence of effective campaigns to promote physical activity at home, there are concerns regarding the independence and mental health of older people [27]. Japanese academic societies are introducing in-home exercises in response to this situation [28]. Additionally, decreased activity and social isolation may increase the risk of cardiovascular disease, stroke, dementia, depression, anxiety, chronic health conditions, and other physical and mental health issues, including suicide attempts [10]. Moreover, changes in care for chronic diseases among users are inevitable, even though nurses play a role in home-visit services. Not only in the LTCI system has a reduction in the use of medical outpatient visits has been reported [29,30]. Future studies will have to track how this lost period affects the health of older adults with disability in the long term. On the other hand, the change in services can affect family caregivers of older adults. In general, outpatient services play an important role in providing respite for users’ families [31]. Without these services, family caregivers might have to face this extra burden. The caregiving burden has been found to be associated with deterioration of caregivers’ health or quitting of community-dwelling in previous studies [32]. These effects will need to be examined over the long term.

Furthermore, it was unknown whether the change in trend of LTCI service use Pre- and Post-COVID-19 occurred because users refrained from using the services, or because the facilities were closed. The influence from both the users’ side and the facility side can be considered. For example, the providers might prioritize services for severely disabled users when considering the necessity of care. However, this is beyond the scope of the present study. Further verification and discussion on countermeasures will be necessary to determine the causes for reluctance to use services. Further, there are no findings on how to meet the needs among mildly disabled persons who are not using services. There is a need for further investigation into how the lost services could be replaced. In the seven prefectures where the state of emergency was initially declared, reports indicated that LTCI service users avoided outpatient services as part of refraining from going out [33]. As for the concern about the facility side, during the pandemic managers refrained from providing services to secure both users and staffs from outbreak. Moreover, preventive measures were difficult to implement due to the unavailability of protective materials and the lack of information regarding COVID-19. Although LTCI service use increased in June 2020, there is still a risk of a second wave of infection. Even if the severity is less than in May 2020, countermeasures should be taken using lessons from the past. Currently, it is expected that the pandemic will be prolonged, and there are concerns that the service staff and facility capacities will be exhausted. Although the Ministry of Health, Labor and Welfare has implemented measures against infection in long-term care establishments [34], the Centers for Medicare & Medicaid Services in the US recommended that staff in LTC facilities and LTC providers reduce their burden when facing this crisis [35]. It is anticipated that a new lifestyle called “With COVID-19” will be established to prevent the spread of infection while supporting daily life. As part of this initiative, technology designed to enhance social contact has been well-developed. While these efforts are still developing, they will eventually be broadly introduced [14,36]. In this context, it is necessary to determine the losses associated with quality of life and health because of the pandemic. Besides, how to maintain physical activity and social interaction while assessing patients’ prognoses should be considered.

### Limitations

The present study has several limitations. First, regarding the representativeness of the collected data, we analyzed insurance claim data collected via a specific system supported by SMS. The data did not include all LTCI services in Japan. However, the SMS system was used by 20.1% of offices nationwide. The offices using the system were spread throughout the country, and the data allowed us to gain insight into nationwide trends. Second, the claim data in the present study only included the results of those using the services. Non-service users among older adults with disabilities were thus excluded. This study described the nationwide trends before and after the spread of COVID-19 in Japan. However, other factors may have influenced the use of LTCI services. Finally, this study merely described the change in use of LTCI services during the COVID-19 pandemic. The influences on this change, such as physical decline or family caregivers’ burden, were not investigated and can only be estimated. Hence, these issues need to be verified in future studies.

## 5. Conclusions

In this study, we described changes in the use of LTCI services for older adults living at home during the COVID-19 pandemic in Japan. The use of outpatient services was reduced; however, home-visit services were mostly maintained. The decrease in use was notable in the seven prefectures where the infection spread rapidly. There are concerns that older adults may become less active and socially isolated due to such changes in LTC service usage, as long term consequences. Further, several adverse events are also expected in the future. It is therefore necessary to conduct future studies and implement sustainable measures from a long-term perspective.

## Figures and Tables

**Table 1:** The number of users of long-term care insurance services by COVID-19 and demographic characteristics.

	Pre-COVID-19						Post-COVID-19					
	2019						2020					
	Jul	Aug	Sep	Oct	Nov	Dec	Jan	Feb	Mar	Apr	May	Jun
Users (persons)												
All services	636,974	642,056	650,204	662,000	667,739	674,120	677,402	683,449	687,510	690,954	690,401	711,852
Home-visit care	213,939	215,423	217,854	221,429	223,429	226,656	227,786	229,238	232,232	234,976	235,575	240,359
Home-visit bathing	9424	9407	9357	9532	9675	9892	9871	9986	10,195	10,811	11,073	11,218
Home-visit nursing	94,425	96,528	98,430	101,313	102,993	104,870	106,091	107,968	110,139	112,393	113,882	119,228
Home-visit rehabilitation	11,258	11,301	11,369	11,666	11,814	11,964	12,045	12,150	12,203	12,475	12,307	12,977
Outpatient care	184,758	185,869	188,248	191,633	193,701	195,007	195,757	197,469	195,610	193,305	190,433	198,784
Outpatient rehabilitation	40,094	40,237	40,695	41,472	41,763	41,871	41,968	42,350	41,899	41,434	40,415	42,338
Male (%)	36.4	36.4	36.4	36.4	36.4	36.4	36.5	36.5	36.5	36.5	36.6	36.6
Age (%) 65–74	13.5	13.6	13.7	13.8	13.9	14.1	12.9	13.0	13.2	13.3	13.4	13.5
75–84	36.5	36.7	36.9	37.1	37.4	37.6	33.9	34.1	34.3	34.4	34.5	34.8
85 +	50.0	49.7	49.3	49.1	48.7	48.3	53.2	52.9	52.6	52.3	52.0	51.7
Care need level (%) CL1	31.4	31.4	31.4	31.4	31.5	31.5	31.6	31.5	31.3	31.0	30.9	31.2
CL2	29.7	29.7	29.8	29.7	29.7	29.8	29.8	29.8	29.8	29.7	29.7	29.7
CL3	17.8	17.8	17.7	17.8	17.7	17.7	17.7	17.7	17.8	17.8	17.9	17.8
CL4	12.5	12.5	12.5	12.5	12.4	12.4	12.4	12.4	12.5	12.6	12.7	12.6
CL5	8.6	8.6	8.6	8.6	8.6	8.6	8.6	8.6	8.7	8.8	8.8	8.7
Seven prefectures (%)	54.8	54.9	55.0	55.1	55.1	55.2	55.3	55.4	55.3	55.0	54.9	55.2

CL: Care level. Seven prefectures include Tokyo, Kanagawa, Saitama, Chiba, Osaka, Hyogo, and Fukuoka.

**Table 2:** The number of users of long-term care insurance services by COVID-19 spread.

		Trend Pre-COVID-19		Change in Level Post-COVID-19		Change in Trend at the Post-COVID-19	
		β_T_	*p*-Value	β_Xt_	*p*-Value	β_TXt_	*p*-Value
	All	0.0120	<0.001	0.0461	<0.001	−0.0038	<0.001
Sex	Male	0.0121	<0.001	0.0413	<0.001	−0.0033	<0.001
	Female	0.0119	<0.001	0.0503	<0.001	−0.0041	<0.001
Age	65–74	0.0195	<0.001	−0.0654	<0.001	−0.0024	0.031
	75–84	0.0180	<0.001	−0.0500	<0.001	−0.0047	<0.001
	85+	0.0053	<0.001	0.1338	<0.001	−0.0028	<0.001
Care need level	CL1	0.0131	<0.001	0.1206	<0.001	−0.0088	<0.001
	CL2	0.0122	<0.001	0.0625	<0.001	−0.0048	<0.001
	CL3	0.0111	<0.001	0.0100	0.490	−0.0013	0.172
	CL4	0.0099	<0.001	−0.0672	<0.001	0.0038	0.001
	CL5	0.0120	<0.001	−0.0305	0.143	0.0013	0.366
Area	Seven prefectures	0.0133	<0.001	0.0857	<0.001	−0.0064	<0.001
	Other prefectures	0.0103	<0.001	−0.0005	0.955	−0.0007	0.241
Service type	Home-visit care	0.0119	<0.001	0.0148	<0.001	−0.0015	<0.001
	Home-visit bathing	0.0100	<0.001	−0.2924	<0.001	0.0188	<0.001
	Home-visit nursing	0.0213	<0.001	−0.0275	<0.001	0.0007	<0.001
	Home-visit rehabilitation	0.0133	<0.001	−0.0006	<0.001	−0.0007	<0.001
	Outpatient care	0.0118	<0.001	0.1827	<0.001	−0.0129	<0.001
	Outpatient rehabilitation	0.0099	<0.001	0.1826	<0.001	−0.0128	<0.001

CL: Care level. Seven prefectures include Tokyo, Kanagawa, Saitama, Chiba, Osaka, Hyogo, and Fukuoka.

## Appendix A

**Table A1:** The fluctuation of person counts during a month during the COVID-19 pandemic.

	Trend Pre-COVID-19			Change in Level Post-COVID-19			Change in Trend at the Post-COVID-19		
	Est.	95% CI		Est.	95% CI		Est.	95% CI	
All	7845.0	7078.6	8611.4	−6751.0	−12,521.4	−980.5	−2229.3	−6010.7	1552.2
Home-visit care	2605.1	2270.5	2939.7	−1589.4	−3055.9	−122.9	−187.4	−841.9	467.1
Home-visit bathing	94.8	30.6	159.1	−112.1	−435.1	210.9	208.4	120.0	296.8
Home-visit nursing	2128.7	2031.2	2226.2	−1713.4	−3053.6	−373.2	319.4	−420.0	1058.7
Home-visit rehabilitation	153.3	117.8	188.8	−125.0	−325.9	75.9	1.1	−123.9	126.1
Outpatient care	2232.2	1899.4	2564.9	−1864.3	−5232.1	1503.5	−2468.7	−4542.0	−395.4
Outpatient rehabilitation	406.9	311.0	502.7	−396.3	−1219.8	427.2	−533.1	−1016.7	−49.6

Est.: Estimates to show the fluctuation of person counts during a month. 95% CI: 95% confidential interval.

**Table A2:** Influence on the number of users of long-term care insurance services by COVID-19, excluding June 2020.

		Trend Pre-COVID-19		Change in Level Post-COVID-19		Change in Trend at the Post-COVID-19	
		β_T_	*p*-Value	β_Xt_	*p*-Value	β_TXt_	*p*-Value
	All	0.0120	<0.001	0.1002	<0.001	−0.0071	<0.001
Sex	Male	0.0121	<0.001	0.0949	<0.001	−0.0066	<0.001
	Female	0.0119	<0.001	0.1032	<0.001	−0.0073	<0.001
Age	65–74	0.0195	<0.001	−0.0234	0.248	−0.0050	<0.001
	75–84	0.0180	<0.001	0.0108	0.388	−0.0084	<0.001
	85+	0.0053	<0.001	0.1841	<0.001	−0.0059	<0.001
Care need level	CL1	0.0131	<0.001	0.2096	<0.001	−0.0142	<0.001
	CL2	0.0122	<0.001	0.1140	<0.001	−0.0080	<0.001
	CL3	0.0111	<0.001	0.0413	0.018	−0.0033	0.005
	CL4	0.0099	<0.001	−0.0421	0.043	0.0022	0.104
	CL5	0.0120	<0.001	−0.0159	0.525	0.0004	0.819
Area	Seven prefectures	0.0133	<0.001	0.1536	<0.001	−0.0105	<0.001
	Other prefectures	0.0103	<0.001	0.0345	0.002	−0.0029	<0.001
Service type	Home-visit care	0.0119	<0.001	0.0341	0.007	−0.0027	0.001
	Home-visit bathing	0.0099	<0.001	−0.3310	<0.001	0.0212	<0.001
	Home-visit nursing	0.0213	<0.001	0.0354	0.056	−0.0032	0.009
	Home-visit rehabilitation	0.0133	<0.001	0.0914	0.098	−0.0063	0.081
	Outpatient care	0.0118	<0.001	0.2890	<0.001	−0.0194	<0.001
	Outpatient rehabilitation	0.0099	<0.001	0.2927	<0.001	−0.0196	<0.001

CL: Care level. Seven prefectures include Tokyo, Kanagawa, Saitama, Chiba, Osaka, Hyogo, and Fukuoka.

## References

[1] Infectious Disease Surveillance Center, National Insitute of Infectous Disease Information of New Coronavirus. https://www.niid.go.jp/niid/ja/from-idsc/2482-corona/9305-corona.html.

[2] Japan Cabinet Secretariat Japan’s Response to the Novel Coronavirus Disease: Declaration of a State of Emergency. https://corona.go.jp/news/news_20200421_70.html.

[3] Wu Z., McGoogan J.M. (2020). Characteristics of and Important Lessons from the Coronavirus Disease 2019 (COVID-19) Outbreak in China: Summary of a Report of 72 314 Cases from the Chinese Center for Disease Control and Prevention. JAMA.

[4] Kawana A., Mikasa K., Izumikawa K. (2020). COVID-19. Nihon Naika Gakkai Zasshi.

[5] McMichael T.M., Currie D.W., Clark S., Pogosjans S., Kay M., Schwartz N.G., Lewis J., Baer A., Kawakami V., Lukoff M.D. (2020). Epidemiology of Covid-19 in a Long-Term Care Facility in King County, Washington. N. Engl. J. Med..

[6] Campbell J.C., Ikegami N. (2000). Long-term care insurance comes to Japan. Health Aff..

[7] Ministry of Health, Labor and Welfare (2018). Report for Provision of Long-Term Care Insurance (Kaigo Hoken Jigyo Jokyo Houkoku). https://www.mhlw.go.jp/topics/kaigo/osirase/jigyo/18/index.html.

[8] Hoogendijk E.O., Smit A.P., van Dam C., Schuster N.A., de Breij S., Holwerda T.J., Huisman M., Dent E., Andrew M.K. (2020). Frailty Combined with Loneliness or Social Isolation: An Elevated Risk for Mortality in Later Life. J. Am. Geriatr. Soc..

[9] Mehrabi F., Béland F. (2020). Effects of social isolation, loneliness and frailty on health outcomes and their possible mediators and moderators in community-dwelling older adults: A scoping review. Arch. Gerontol. Geriatr..

[10] National Academies of Sciences (2020). Social Isolation and Loneliness in Older Adults: Opportunities for the Health Care System.

[11] Gale C.R., Westbury L., Cooper C. (2018). Social isolation and loneliness as risk factors for the progression of frailty: The English Longitudinal Study of Ageing. Age Ageing.

[12] Simard J., Volicer L. (2020). Loneliness and Isolation in Long-term Care and the COVID-19 Pandemic. J. Am. Med. Dir. Assoc..

[13] Plagg B., Engl A., Piccoliori G., Eisendle K. (2020). Prolonged social isolation of the elderly during COVID-19: Between benefit and damage. Arch. Gerontol. Geriatr..

[14] Eghtesadi M. (2020). Breaking Social Isolation Amidst COVID-19: A Viewpoint on Improving Access to Technology in Long-Term Care Facilities. J. Am. Geriatr. Soc..

[15] Jawaid A. (2020). Protecting older adults during social distancing. Science.

[16] Ministry of Health, Labor and Welfare Ethical Guidelines for Medical and Health Research Involving Human Subjects. https://www.mhlw.go.jp/stf/seisakunitsuite/bunya/hokabunya/kenkyujigyou/i-kenkyu/index.html.

[17] Ministry of Health, Labor and Welfare How is the Certification of Need for Nursing Care Done?. https://www.mhlw.go.jp/stf/seisakunitsuite/bunya/hukushi_kaigo/kaigo_koureisha/nintei/gaiyo2.html.

[18] Ministry of Health, Labor and Welfare Overview of the 2015 Survey of Long-Term Care Service Facilities and Offices. https://www.mhlw.go.jp/toukei/saikin/hw/kaigo/service15/dl/kekka-gaiyou_03.pdf.

[19] Lopez Bernal J., Cummins S., Gasparrini A. (2017). Interrupted time series regression for the evaluation of public health interventions: A tutorial. Int. J. Epidemiol..

[20] Linden A. (2015). Conducting interrupted time-series analysis for single- and multiple-group comparisons. Stata J..

[21] Prime Minister of Japan and His Cabinet Information Related to COVID-19. https://www.kantei.go.jp/jp/headline/kansensho/coronavirus.html.

[22] Ministry of Health, Labor and Welfare Social Security Council (Kaigo Kyuhu-hi Bunkakai) 188th (held on 15 October 2020) Document 1: Outpatient Care/Community-Based Outpatient Care/Reward for Outpatient Care for Dementia—About the Standard. https://www.mhlw.go.jp/content/12300000/000683014.pdf.

[23] Ministry of Health, Labor and Welfare Home-Visit Care and Bathing. https://www.mhlw.go.jp/content/12300000/000660330.pdf.

[24] Meléndez J.C., Tomás J.M., Navarro E. (2011). Everyday life activities and well-being: Their relationships with age and gender in the elderly. Ann. Psychol..

[25] Lim W., Liang C., Assantachai P., Auyeung T.W., Kang L., Lee W., Lim J., Sugimoto K., Akishita M., Chia S. (2020). COVID-19 and older people in Asia: Asian Working Group for Sarcopenia calls to actions. Geriatr. Gerontol. Int..

[26] Smith B.J., Lim M.H. (2020). How the COVID-19 pandemic is focusing attention on loneliness and social isolation. Public Health Res. Pract..

[27] Goethals L., Barth N., Guyot J., Hupin D., Celarier T., Bongue B. (2020). Impact of Home Quarantine on Physical Activity Among Older Adults Living at Home During the COVID-19 Pandemic: Qualitative Interview Study. JMIR Aging.

[28] The Japan Geriatrics Society COVID-10, Practice Caution for Older People. https://www.jpn-geriat-soc.or.jp/coronavirus/index.html.

[29] Ministry of Health, Labor and Welfare Impact of the New Coronavirus Infection on the Medical Insurance System. https://www.mhlw.go.jp/content/12401000/000693625.pdf.

[30] KENPOREN National Federation of Health Insurance Societies Statistical Data: Recent Trends in Health Insurance Association Medical Expenditures on September 2020. https://www.kenporen.com/toukei_data/pdf/chosa_r02_11_03.pdf.

[31] Tatematsu M. (2014). On Problem of Respite Care Services Provision Appeared from Investigation to Cross Home Caregivers’ Daily-Care-Stresses and Use Effect of Respite Care Services—A Study on the Way Forward Nursing Home to Create a Sense of Community for Frail Elderly People (Part 2). J. Japan Soc. Home Econ..

[32] Etters L., Goodall D., Harrison B.E. (2008). Caregiver burden among dementia patient caregivers: A review of the literature. J. Am. Acad. Nurse Pract..

[33] Japan Federation of Kaigo Business Providers Report of “Urgent Survey” on the Impact of the New Coronavirus Infection on Business. http://kaiziren.or.jp/wp/wp-content/uploads/2020/04/kinkyuutyousa20200422.pdf.

[34] Ministry of Health, Labor and Welfare Merasurement to New Coronavirus Infections at Nursing Care Providers. https://www.mhlw.go.jp/stf/seisakunitsuite/bunya/0000121431_00089.html.

[35] Levitt A.F., Ling S.M. (2020). COVID-19 in the Long-Term Care Setting: The CMS Perspective. J. Am. Geriatr. Soc..

[36] Middleton A., Simpson K.N., Bettger J.P., Bowden M.G. (2020). COVID-19 Pandemic and Beyond: Considerations and Costs of Telehealth Exercise Programs for Older Adults With Functional Impairments Living at Home-Lessons Learned From a Pilot Case Study. Phys. Ther..

